# Self-Administered Mind-Body Practices for Reducing Health Disparities: An Interprofessional Opinion and Call to Action

**DOI:** 10.1155/2016/2156969

**Published:** 2016-09-08

**Authors:** Patricia A. Kinser, Jo Lynne W. Robins, Saba W. Masho

**Affiliations:** ^1^Virginia Commonwealth University School of Nursing, Richmond, VA, USA; ^2^Department of Family Medicine and Population Health, Division of Epidemiology, Virginia Commonwealth School of Medicine, Richmond, VA, USA

## Abstract

Health disparities (HD) continue to persist in the United States which underscores the importance of using low-cost, accessible, evidence-based strategies that can improve health outcomes, especially for chronic conditions that are prevalent among underserved minority populations. Complementary/integrative health modalities, particularly self-administered mind-body practices (MBP), can be extremely useful in reducing HD because they are intrinsically patient-centered and they empower patients to actively engage in self-care of health and self-management of symptoms. Interprofessional healthcare providers and patients can engage in powerful partnerships that encompass self-administered MBP to improve health. This is a call to action for interprofessional researchers to engage in high-quality research regarding efficacy and cost-effectiveness of self-administered MBP, for practitioners to engage patients in self-administered MBP for health promotion, disease prevention, and symptom management, and for healthcare institutions to integrate self-administered MBP into conventional health practices to reduce HD in their communities.

## 1. Introduction

Racial/ethnic health disparities (HD) or inequalities in access to healthcare and in quality of healthcare are well documented across a range of health conditions, services, and settings in the United States [1]. Despite widespread awareness and recommendations regarding the importance of increasing access and culturally appropriate health delivery systems, HD continue to persist. Health disparities are defined by the Institute of Medicine (IOM) as differences in the quality of healthcare due to racial or ethnic differences, typically due to discrimination at the patient-provider level or at the level of health systems [2]. Although HD also can be defined more broadly as being due to disadvantages which occur beyond racial/ethnic status such as socioeconomic status, gender, age, disability, environment, and geographic location [3], this paper is focused in scope on addressing racial/ethnic minority-based HD. Immediate, creative, accessible, cost-effective solutions that are interprofessional in nature and that complement other on-going efforts are greatly needed for addressing HD.

The use of complementary/integrative health practices may provide a creative, accessible, cost-effective solution to complement other on-going efforts for addressing HD. As a subcategory of complementary/integrative health approaches, self-administered mind-body practices (MBP) typically involve an initial education or training session by a clinician or trainer after which patients continue using them on their own as needed or on a regular schedule. For example, a patient is taught a simple breathing technique to enhance relaxation when feeling anxious and the patient then practices the technique on his/her own as needed. Self-administered MBP include a variety of practices such as yoga, tai chi, qi gong, meditation/mindfulness, progressive relaxation, guided imagery, and sensory-therapies (e.g., art, music, play, aroma, and similar therapies) [4, 5]. MBP have been shown to enhance resilience to stress, improve symptom management, and maintain health in various populations [4]. When MBP are self-administered and do not require regular treatment from or payment to a clinician/specialist, these practices may provide a cost-effective method for empowering patients to promote and maintain health and/or self-manage disease symptoms [6, 7]. Self-administered MBP may be relevant for decreasing the incidence and sequelae of disparity-related biopsychosocial stress in underserved minority populations who have limited resources yet high levels of stress vulnerability and related morbidity and mortality.

The sources of disparities in the healthcare system are typically attributed to multilevel factors (e.g., those which occur at the patient level, the provider level, and the care-system level) and the use of multilevel strategies is recommended in order to address HD and improve health outcomes [2]. As such, we propose an interprofessional multilevel solution based on the integration of complementary health practices into conventional health settings, particularly the teaching and recommending of self-administered MBP by healthcare providers to patients with the goal of enhancing stress resilience and improving health. The goal of this paper is to describe the current use of complementary/integrative approaches in the United States, to discuss how MBP can be based upon patient-centered interprofessional collaboration, and to propose that self-administered MBP may be powerful tool for addressing provider level factors (e.g., enhancing patient-provider partnerships) and system-level factors (e.g., addressing limitations in healthcare access) to ultimately reduce HD in racial/ethnic minority populations [2].

## 2. Use of Complementary/Integrative Approaches

The popularity of complementary/integrative health approaches, such as MBP, by consumers has grown substantially over the last decade. Approximately 34–36% of the US population has used at least one complementary approach in the prior two years [8–10], with the most common therapies including natural products, relaxation and breathing exercises, meditation, yoga, and massage. Data from the 2012 National Health Information Survey (NHIS) suggests that approximately 33.2% of the population in the United States uses some form of complementary health approach and the most commonly stated reasons for use of these approaches are for pain management and stress relief [9]. Approximately 9.5% of the US population uses a mind-body complementary health approach, such as yoga, chiropractic manipulation, meditation, and massage therapy [9]. Hispanics and non-Hispanic Blacks constitute 41.3% of all complementary health practice users (22% by Hispanics; 19.3% by non-Hispanic Blacks) [9]. Of particular interest for HD researchers, NHIS data suggests that 5.4% of the US population or 12.3 million use complementary approaches as alternative therapies to conventional medications in order to save money [11]. The people who do so are more likely to be Hispanic (6.9%), be uninsured (11.9%), and have an income below the federal poverty line (7.6%) [11]. Further, a substantial percentage of the population (47.6%) does not inform their healthcare providers about use of these approaches [12].

Many factors likely influence the use of complementary/integrative approach by minority populations which face HD. First, individuals may have past experiences in which conventional medical care did not meet specific needs, was inaccessible, or was cost-prohibitive; thus the individuals turn to complementary approaches for symptom management or health maintenance [10, 13, 14]. Second, there may be a community-wide historic inherent distrust of conventional healthcare providers or institutions (e.g., Tuskegee Syphilis Study) or a history of overt negative experiences with conventional healthcare. For example, data from the National Survey of Midlife Development in the United States of Black adults suggest that a history of racial discrimination, even in nonmedical contexts, is associated with a higher likelihood of using complementary approaches as a means of healthcare [15]. A third motivation for turning to complementary approaches includes the desire to embrace practices which avoid conventional treatment-related side effects [16]. Fourth and finally, many racial and ethnic groups have long used complementary/integrative health approaches as part of their holistic culturally based understanding of health [16]. Of importance, NHIS data suggest that non-Hispanic White adults are more likely to choose complementary approaches which are provider-based, such as chiropractic manipulation, massage, and acupuncture, whereas non-White adults are more likely to choose self-administered therapies, such as relaxation practices, yoga, meditation, and tai chi [10].

## 3. Interprofessional Approach

An interprofessional approach to healthcare delivery has received attention as a means to improve patient-centered care. An interprofessional approach entails the collaboration of professionals from different disciplines, working and communicating with each other, providing knowledge and skills, to augment and support the contributions of others and optimize patient care [17]. The IOM report,* The Future of Nursing: Leading Change, Advancing Health*, strategically called for and initiated a movement towards transforming the healthcare system to integrate interprofessional collaboration and coordination as the standard of care [18].

MBP lends itself for an interprofessional collaboration and can be integrated into any system of care. Healthcare professionals including physicians, nurses, psychologists, social workers, and other therapists can work together to teach or recommend self-administered MBP as needed. In fact, nurses, nurse practitioners, and therapists are strategically positioned to coordinate or educate patients on self-administered MBP. Interprofessional teams are positioned to connect a multitude of providers and promote a collaborative practice that hinges on each profession recognizing and utilizing each other's expertise to provide an effective patient-centered care.

## 4. Self-Administered MBP: Patient and Provider Level

Given that ethnic and racial minority populations tend to choose self-administered therapies, it is relevant to explore how these MBP may enhance health. Self-administered MBP are focused on the integration of the mind-body to affect physical and mental functioning, enhance stress resilience, and promote overall health [4]. There are a wide variety of self-administered MBP, yet most of them share basic, easily accessible processes for achieving relaxation, enhanced attention, and mindful awareness. In self-administered MBP, breathing and gentle physical movement are paired with focused attention and mindfulness (or present moment awareness) [19]. Clinical trials evaluating various forms of self-administered MBP, including relaxation practices, yoga, tai chi, and meditation, have been shown to help various populations maintain health through self-regulation and stress resilience [20–22]. Further, self-administered MBP may help participants manage acute or chronic conditions. In particular, trials of yoga, tai chi, and mindfulness meditation have been shown to decrease physical and psychological symptoms associated with the following conditions: depression [23–25], hypertension [26], cardiometabolic conditions [27], chronic inflammatory conditions [28, 29], PTSD [30], menopause [31], chronic pain [32–35], and substance abuse [36], among others.

The process of self-administration of a MBP allows patients to self-manage an illness or maintain health. Self-management of health or illness is based upon the patient's active participation in healthy self-care activities [37]. This act of self-administration and self-management can be an incredibly empowering experience for the patient, particularly when the efforts are supported by their healthcare providers [38, 39]. Self-management approaches have successfully helped patient address many chronic diseases [37, 38, 40, 41]. We contend that self-administration of a MBP might be a key component of health or illness self-management.

Healthcare providers may incorporate information about self-administered MBP into clinical practice in multiple ways. First, providers may wish to refer patients to community resources, web-based resources, or printed literature about MBP. Second, providers who are personally familiar with MBP and are comfortable with the content may provide informal teaching and education during patient interactions. Third, providers may offer more formalized sessions or workshops during which patients may learn about self-administered MBP; this may require formal institutional support of MBP, addressed in the next section.

With basic training, healthcare providers can be in an excellent position to educate on and encourage the use of basic self-administered MBP for enhancing stress resilience and health. Unfortunately, many healthcare providers have not received formal training about complementary/integrative health practices during their educational experiences; however many are seeking out resources for their own self-care or to enhance their patient care [22, 42]. Increasingly, nursing and medical schools are incorporating information about complementary/integrative approaches into curricula [43, 44] and continuing education programs opportunities are available for practicing clinicians. However, educational opportunities for healthcare providers about self-administered MBP should become more available because, given simple tools, healthcare providers may quickly and easily teach or refer MBP to patients who would benefit from enhanced self-care and improvements in physical and mental health [45]. Providers may find that partnering with patients in this way could create powerful health promoting patient-provider relationships as well as help alleviate issues of alienation and mistrust in racially and ethnically diverse underserved populations, potentially leading to greater engagement in self-management of health and illness in these populations.

## 5. Self-Administered MBP: Health System and Policy Levels

At the health system level, limitations on access to care, including limited availability of facilities or services in certain areas and limited time available for patients to see healthcare providers, are important contributors to HD. These circumstances present an important opportunity for engaging underserved populations in self-care. Healthcare providers and healthcare systems may consider the low cost of brief education sessions and encouragement regarding self-administered MBP to enhance the health of populations in need, particularly when other resources are limited. In order to do so, policies that support and encourage the use of MBP are required. Financial incentives should be available to reduce barriers to therapies which may be beneficial to patients and to enhance the time available for patient-provider communication about these therapies [2]. In those conditions for which evidence is building that MBP can improve health outcomes, discussed above, payment policies should be developed to support provider facilitation for MBP among patient populations.

Another systems-level strategy for encouraging the use of MBP is to encourage its use and target providers in facilities where underserved minority patients are likely to seek care. For example, community-based health centers might introduce key concepts of MBP in structured classes and provide resources for individuals to self-administer the MBP in the future. It has been reported that as many as two-thirds of community health centers are already actively using complementary/integrative approaches [46] and numerous academic medical centers have incorporated integrative health into healthcare and educational programs [45, 47]. Therefore, models exist and should be replicated by interprofessional groups interested in incorporating self-administered MBP therapies into community health centers and academic medical centers.

Certainly, large-scale research studies are required to analyze implementation costs into health systems and to evaluate cost versus benefits for various therapies. However, numerous high-quality studies report that complementary/integrative health therapies may be cost-effective and even provide cost-savings to institutions [6, 48]. For example, the systematic review of economic evaluations by Herman and colleagues (2012) suggests that, in high-quality studies which compared a complementary therapy with usual care, approximately 30% of cost-effectiveness comparisons showed cost-savings, as opposed to only 9% of economic comparisons across allopathic therapies [6]. At a research and policy level, innovative research questions and designs that foster a better understanding of the impact of historical and sociopolitical contributors on psychosocial risk factors, health disparities, and health outcomes are needed. Priority should be given to high-quality research on complementary/integrative health therapies that have some demonstrated intervention efficacy with high potential benefits and that focus on highly prevalent conditions that cause great burdens of suffering [45].

## 6. Summary

We propose that self-administered MBP therapies can be useful in reducing HD and call interprofessional providers and researchers to engage in this important area of research and practice. Continued HD in the United States underscore the significance of encouraging the use of simple, low-cost, highly accessible, evidence-based strategies that can improve health outcomes, particularly for health promotion and prevention of chronic conditions that are prevalent among underserved minority populations. While the focus of this paper has been on HD in racial and ethnic minorities, we recognize that HD exist in any group that experiences greater morbidity and mortality as a result of disadvantage or discrimination and believe research is warranted regarding how MBP may provide opportunities to decrease HD along multiple other dimensions, such as income, education, gender, disability, and insurance status [49]. Although research is required to more fully evaluate efficacy, efficiency, and cost-savings of using complementary/integrative health therapies, the preliminary evidence suggests that the use of self-administered MBP in underserved populations is highly relevant.

The integration of MBP into daily life and care practices may require a shift in perspective for healthcare providers and patients. However, the goal of reducing and ultimately eliminating HD requires that healthcare providers, patients, and policy-makers creatively work together on health promotion, disease prevention, and symptom management. Discussions of the potential benefits and basic techniques of MBP along with the provision of conventional healthcare are well within the scope of practice for interprofessional healthcare providers. Given the right tools, patients can engage in powerful partnerships with their care providers and healthcare systems that encompass self-care practices to improve quality of life and health.
